# Ramadan Fasting and Exercise Performance

**DOI:** 10.5812/asjsm.34859

**Published:** 2010-09

**Authors:** S Javad Fallah

**Affiliations:** 1 Sports Medicine Research Center, Tehran University of Medical Sciences, Tehran, I.R. Iran; 2 Faculty of Nutritional Sciences and Dietetics, Tehran University of Medical Sciences, Tehran, I.R. Iran

**Keywords:** Ramadan fasting, Exercise performance, Sport

Most healthy adults in Muslim communities practice fasting during the holy month of Ramadan. This kind of fasting involves daily abstinence from food and water, from sunrise to sunset, a period that lasts approximately 12 to 17 hours, depending on the season and geographical latitude. Ramadan is a lunar month, which lasts 28 to 30 days and its length changes along the solar year.

Ramadan fasting cannot be simply considered as a different diet. The reason is that the pattern and timetable of eating, drinking and sleeping change in addition to alterations in the food composition during Ramadan.

In most Muslim countries semi-professional and professional sport activities and competitions are postponed until after sunset, and sometimes even until late at night in the month of Ramadan. Fasting and rescheduling of the competition timetable may both affect an athlete's performance during this period.

Sport competitions continue throughout the month of Ramadan. For instance, London 2012 Olympic Games will run from 27 of July to 12 of August, while Ramadan will start on July 21 and last until August 20. Moreover, there is an increasing number of ‘Ramadan Cup’ competitions in different Muslim countries.

Currently, there exists a limited knowledge pertaining to the physiological and performance consequences of athletes' fasting in Ramadan. However, the issue has been addressed by many original and review articles recently ^[1]^.

One day of fasting seems to have no or a little effect on performance. However, thirty consecutive days of fasting may affect various performance factors including endurance and cognitive functions.

Controversy amongst the results of research studies continues to increase due to several reasons such as differences in Ramadan culture, food composition, daily lifestyle, climate, altitude, length of the day and the choice of performance tests.

It is noteworthy that several questions remain in this regard, needed to be investigated. Some of them are as follows:

- What is the best time for training and competition for athletes in different sports during day and night?
- How can the negative effects on performance — if there are any – be minimized? What is the best eating and hydration strategy?
- What is the difference between a bodybuilder and a distance runner in terms of the implications of Ramadan fasting, and what is the best nutritional and training strategy?
- What is the impact of fasting on special populations such as older or younger athletes?
- How does Ramadan fasting affect the brain and its functions?
- Are there any differences between males and females in response to Ramadan fasting?

According to the aforementioned questions, it seems that we are still at the beginning of a long journey to answer all of the relevant questions.

Asian Journal of Sports Medicine aims to provide a platform for all researchers around the world to share the results of their studies on various aspects of shortterm fasting, particularly Ramadan-style fasting, and its probable effects on sports and exercise performance. All accepted manuscripts will be published in a special issue.

We hope that this will help in promoting knowledge and understanding of Ramadan fasting and its effects on exercise and sports performance, and will help in making clearer conclusions and wiser recommendations for athletes and for organizers of sports events taking place during Ramadan.
